# Missed Lung Cancers on the Scout View: Do We Look Every Time?

**DOI:** 10.1155/2013/760543

**Published:** 2013-12-29

**Authors:** Sarfraz Ahmed Nazir, Rachel Benamore, Fergus Gleeson

**Affiliations:** ^1^Department of Radiology, Horton Hospital, Oxford University Hospitals NHS Trust, Oxford OX16 9AL, UK; ^2^Department of Radiology, Churchill Hospital, Oxford University Hospitals NHS Trust, Oxford OX3 7LE, UK

## Abstract

Scout views are digital radiographs obtained to aid planning of the subsequent computed tomography (CT) examination. Review of these scout views may provide additional information not demonstrated on the axial images, but such reviews may not necessarily be performed routinely, especially in the context of abdominopelvic CT studies. We illustrate the value of the scout images by presenting a series of representative cases of missed pulmonary neoplasms in five patients who originally underwent such examinations.

## 1. Introduction

The “scout” view is an obligatory part of performing computed tomography (CT). It is synonymously known as a scanogram, topogram, localiser, scan projection radiograph, surview, or pilot scan. These images are usually obtained with the patient in the supine position but can also be generated in the prone, oblique, or decubitus orientation. They are projectional overviews generally used as reference images for acquiring an axial CT series. Abdominopelvic CT examinations frequently include the lower chest on the “scout” view, but the most cranial axial CT sections usually start lower than the most cranial portion of the “scout,” potentially leaving lesions if present on the uppermost part of the “scout” not included on the axial sections. As the “scout” views are not of primary interest to the reporting radiologist, they may not be reviewed during reporting on picture archiving and communications systems (PACS) workstations, potentially resulting in a failure to detect abnormalities not included on the axial sections. We present a series of 5 cases that we have encountered in our practice illustrating the absolute necessity to review the scout images when reporting abdominopelvic CT, as summarised in Table 1.

## 2. Case 1

**Figure 1:**
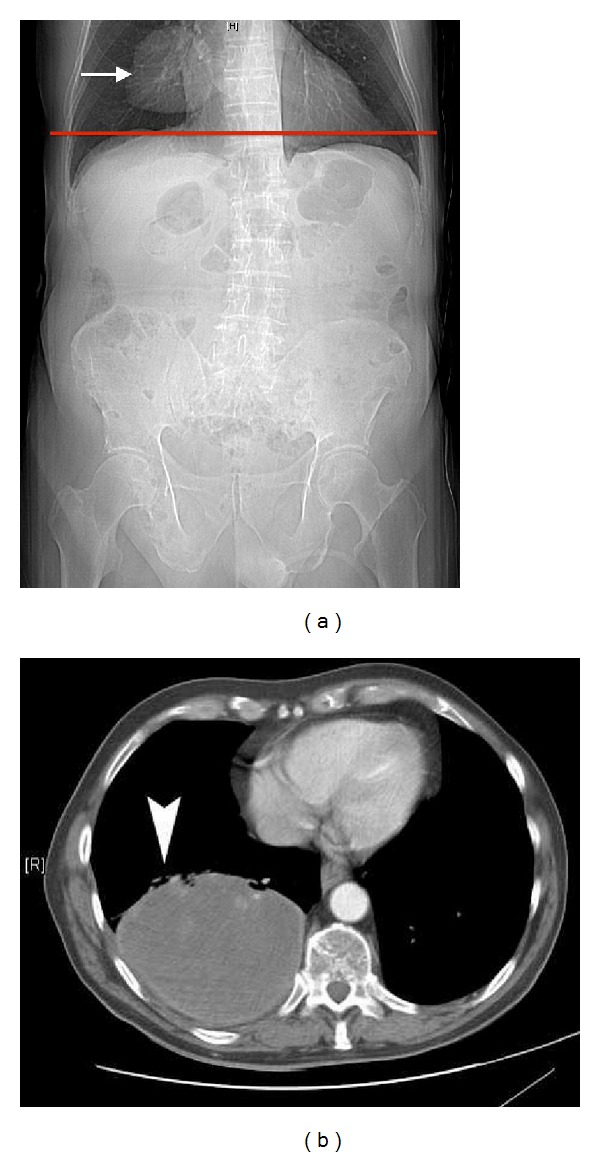
(a) CT scout (AP) view: large right lower lobe mass (white arrow). The level of the first axial image from the CTU is shown by the red line demonstrating that the scan commenced below the level of the lesion. (b) Image from the subsequent CT chest scan confirming a right lower lobe mass (arrowhead).

An 88-year-old patient with a prior history of caecal carcinoma which was resected 2 years earlier presented to his family doctor with persistent macroscopic haematuria. A CT urogram (CTU) reported only marked benign prostatic hypertrophy. A plain chest radiograph performed 7 months later for a persistent cough identified a large right lower lobe mass, measuring 10 cm in size. Review of the CTU identified a 7 cm mass in the right lower lobe on the scout image (Figure 1), but the axial sections had started below the mass. Biopsy confirmed metastatic adenocarcinoma of the caecum.

## 3. Case 2

**Figure 2:**
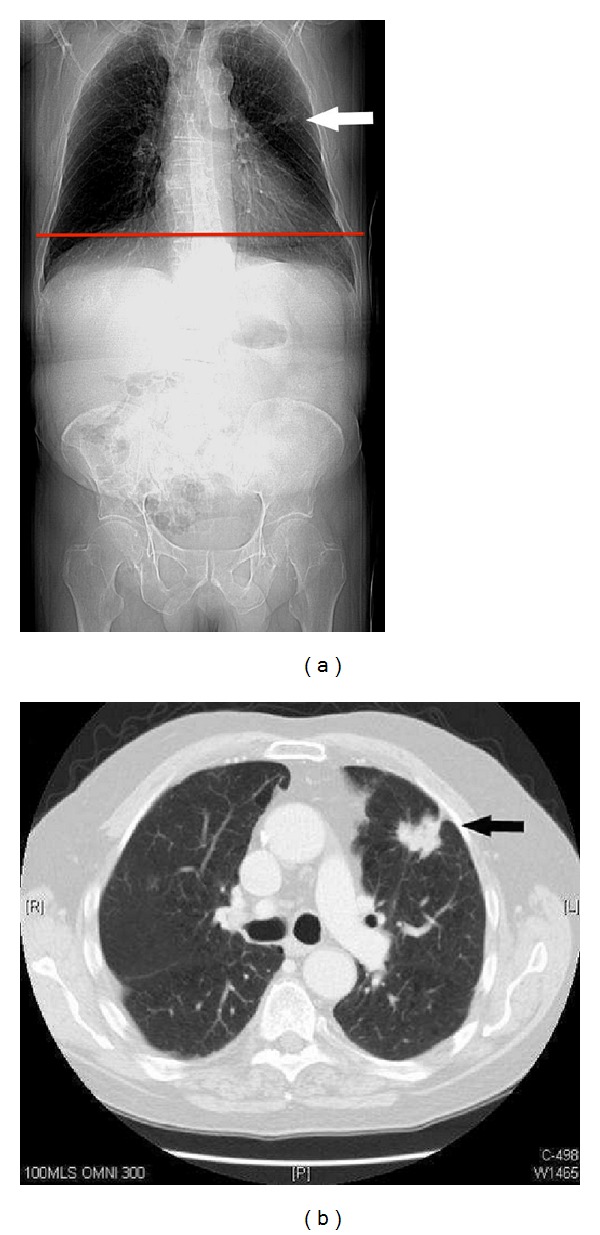
(a) CT scout (AP) view demonstrates a nodule in the left midzone (white arrow). The level of the first axial image from the surveillance abdominopelvic CT is shown by the red line demonstrating that the scan commenced below the level of the lesion. (b) Image from CT chest scan confirmed a soft tissue attenuation spiculated nodule with a pleural tag in the anterior segment of the left upper lobe (black arrow).

A 75-year-old gentleman with prior history of rectal cancer in the setting of ulcerative colitis treated 3 years earlier with a panproctocolectomy underwent a surveillance abdominopelvic CT scan. Axial CT images of the abdomen and pelvis demonstrated no abnormality. A chest radiograph performed 7 months later for chest symptoms revealed a 2 cm left midzone opacity. Review of the prior CT scout confirmed it to be visible. Subsequent CT of the chest and biopsy confirmed it to be a squamous cell bronchogenic carcinoma (Figure 2).

## 4. Case 3

**Figure 3:**
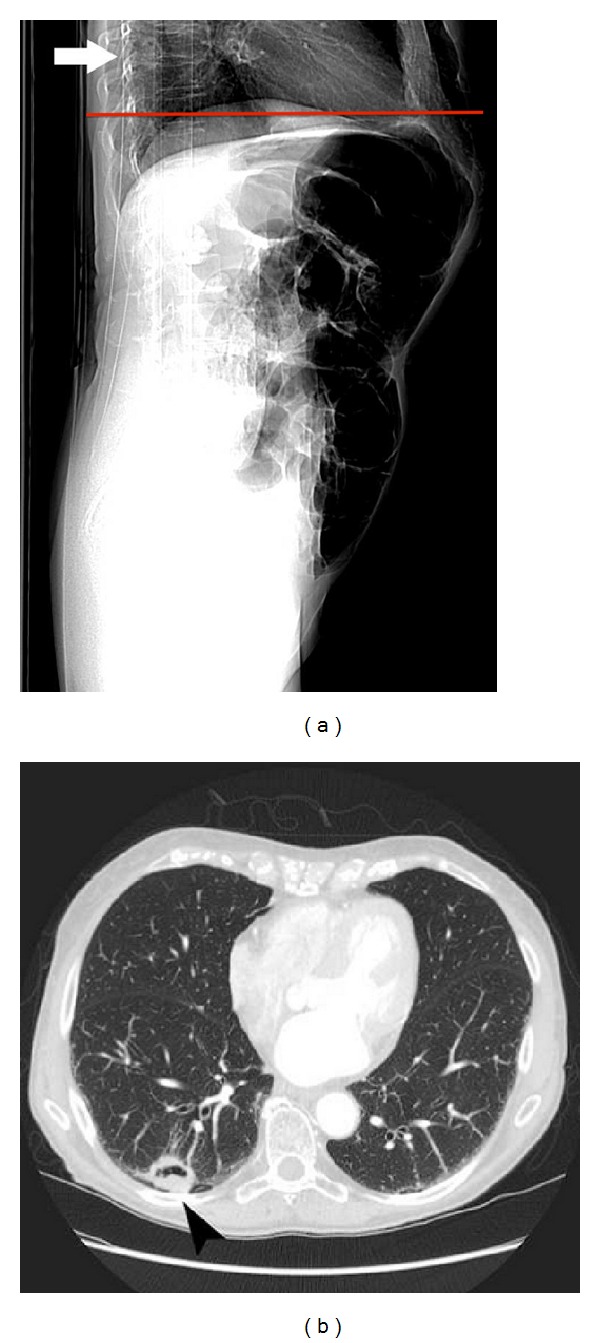
(a) CT scout (AP) view demonstrates a solitary cavitating nodule posteriorly within the right lower lobe (white arrow). The level of the first axial image from the CT colon study is shown by the red line, demonstrating that the scan commenced below the level of the lesion. (b) A selected image from the chest component of the subsequent CT examination demonstrates a cavitating soft tissue attenuation nodule within the posterior basal segment of the right lower lobe (black arrowhead).

A CT colon study was performed on a 75-year-old woman with a history of rectal bleeding. No significant pathology was delineated except for a small right basal nodule, correctly reported as rounded atelectasis. The rectal bleeding persisted and a repeat CT study of the chest, abdomen, and pelvis was performed nearly three months later. No colonic abnormality was seen on the abdominopelvic component but a 3 cm diameter cavitating mass was demonstrated on the chest component, just superior to the previously reported rounded atelectasis. Review of the initial CT demonstrated the mass on the lateral scout view (Figure 3). Resection revealed the mass to be a bronchoalveolar cell carcinoma.

## 5. Case 4

**Figure 4:**
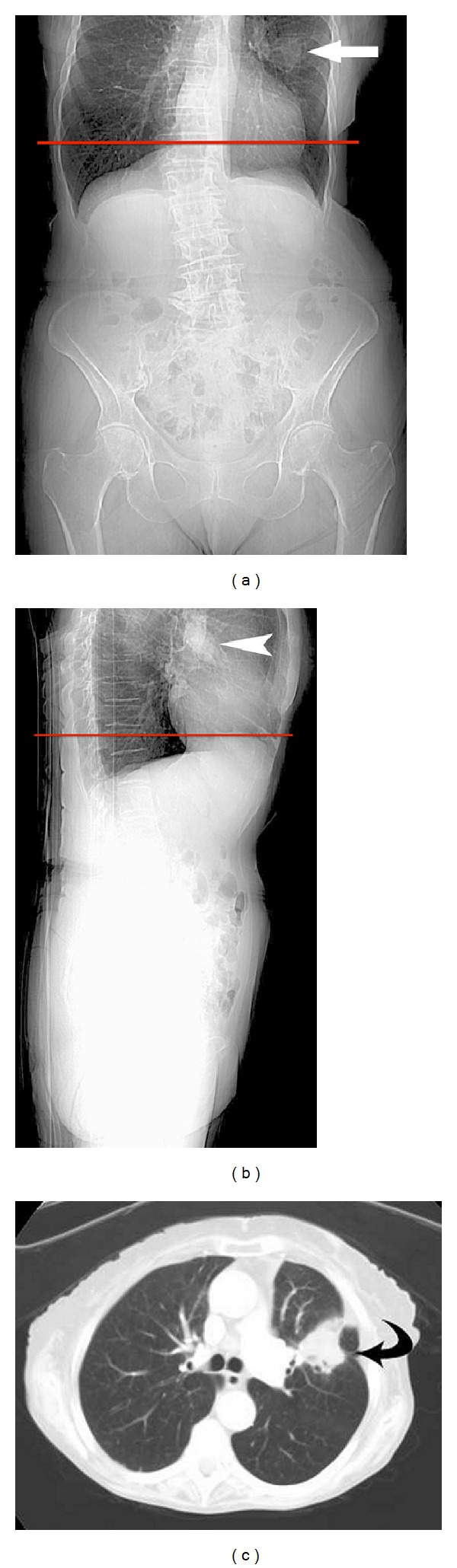
(a) AP and (b) lateral CT scout views demonstrate a left midupper lobe soft-tissue density mass (arrow, arrowhead). The level of the first axial image from the abdominopelvic CT is shown by the red lines, demonstrating that the scan commenced below the level of the lesion. (c) Chest CT axial image demonstrates a mass in the anterior segment of the left upper lobe (curved black arrow).

An 86-year-old lady presented with anaemia, diarrhea, and weight loss. A contrast enhanced CT study of the abdomen and pelvis was reported as normal. A 4 cm diameter mass in the left midzone was reported on a plain chest X-ray performed 8 months later. Review of the prior CT confirmed that the mass was evident on both the AP and lateral scout views (Figure 4). The mass was confirmed to be a nonsmall cell bronchogenic carcinoma by bronchial biopsy.

## 6. Case 5

**Figure 5:**
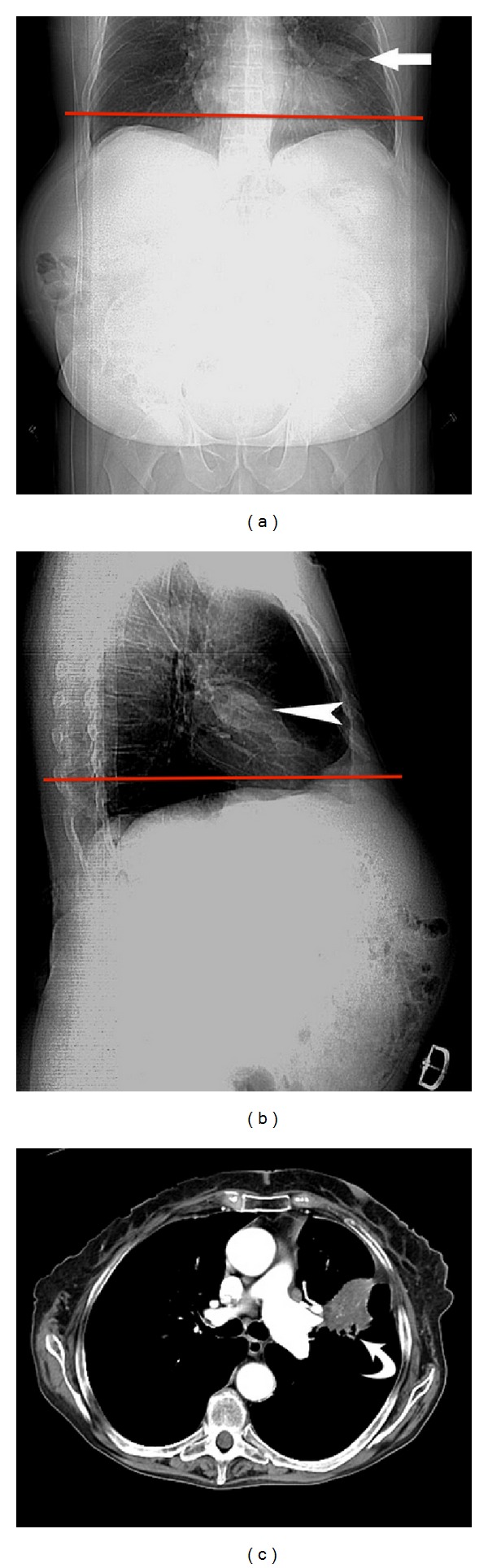
(a) AP and (b) lateral CT scout views demonstrate the lingular mass (arrow, arrowhead). The level of the first axial image from the colonic CT is shown by the red line, demonstrating that the scan commenced below the level of the lesion. (c) Axial chest CT section demonstrating the lingular mass (curved arrow).

A 69-year-old gentleman being investigated for iron deficiency anaemia underwent a CT colon which was reported as normal. A chest radiograph performed 3 months later demonstrated lingular consolidation which failed to resolve on antibiotic treatment. A subsequent thoracic CT examination demonstrated a 4.5 cm lingular mass. Review of the initial CT study confirmed the mass to be visible on the scout images but had not significantly changed in size (Figure 5). The patient was too claustrophobic for PET-CT or MRI imaging and a further CT examination of the chest performed 9 months after the initial CT colon revealed an enhancing mass albeit still unchanged in size. CT guided core biopsy was arranged, which revealed a diagnosis of bronchoalveolar cell carcinoma.

## 7. Discussion

The scout view is a digital X-ray projection radiograph generated by maintaining the CT gantry in a fixed position as the patient is translated through the X-ray beam. Usually obtained with parameters set at a high kilovoltage and low milliampere, its primary role is being a tool for determination of the appropriate anatomical levels and gantry angles whilst acquiring axial CT sections [1, 2].

Whilst not primarily used for diagnostic review, the scout images of an abdominopelvic CT scan may contain diagnostic information not available on the axial CT sections. The scout views of a CT examination are automatically copied as a separate image series to picture archiving and communication system (PACS). Consequently unless reporting radiologists automatically load all images, they have to specifically load and review the scout images, and this may not be routine practice. As shown in the 5 cases reported here, in the case of abdominopelvic CT, this is especially important as the scout may uncover an abnormality not covered on the axial images.

That CT scout images may contain substantial and clinically relevant information not available on the axial CT images, has been reported previously for head and neck imaging.

Sener et al. retrospectively analysed 100 randomly selected CT examinations of the Head and 100 CT examinations of the spine. In 122 patients with demonstrable pathology on the CT sections or the scout image, a total of 154 abnormalities were found, of which 31 (20%) were identified only on the scout images [3]. In a later case series of six paediatric patients, important findings in the upper cervical spine and neck that were not included in the axial slices were clearly visualised on the CT scout view of their CT head examinations [4].

It would have been possible to detect all the abnormalities identifiable on the scout images had the chest been formally examined by CT along with the abdomen, and pelvis CT, and it may be argued that a CT of the chest, abdomen and pelvis should have been routine practice in the two patients with a prior history of colorectal carcinoma. Case 1 was referred by a urological surgeon whose practise does not include routine request for chest CT, and case 2 was a referral from an outside hospital, where the practise is not to routinely include the chest. Both of these cases suggest that scans may be obtained from other practises that might not scan the chest and further strengthen the argument to review the scout images.

In our department, radiologists feedback discrepancies that they come across in their daily practice. Discrepancies, which are raised at multidisciplinary meetings, and complaints that are received from clinicians are also fed into the same system. The process is facilitated by a radiologist filling in an anonymous slip with details of the case and the date of examination.

These are placed in locked boxes, which are emptied regularly by the audit lead radiologist.

The discrepancy convenor reviews the appropriate films and takes notes. The images are then peer reviewed at monthly meetings. The cases presented here were discovered via this process over a one-year period. It is the authors' view that had scout images been reviewed from *all* abdominopelvic CT scans performed over this time, more such cases would have come to light.

In conclusion, we have shown that CT scout images may play a crucial diagnostic role in patients undergoing abdominopelvic CT studies. Overlooking an abnormality on the CT scout view is a potential risk, which can easily be avoided by developing the habit of systematically screening the scout view on every CT examination.

## 8. Teaching Point

Scout views are digital radiographs obtained to aid planning of the subsequent computed tomography (CT) examination and may provide additional information not demonstrated on the axial images. Such reviews should be performed routinely, especially in the context of abdominopelvic CT studies, so that serious pathology is not missed.

## Figures and Tables

**Table 1:** Summary of 5 lung cancers missed on the scout views of abdominopelvic CT scans.

Patient	Age (years)	Initial exam	Delay to diagnosis (days)	Pathology	Outcome
1	88	CT urogram	235	Metastatic colonic adenocarcinoma	Nonoperable radiotherapy
2	75	CT abdomen and pelvis	224	Squamous cell bronchogenic carcinoma	Resection
3	75	CT colon	79	Bronchoalveolar cell carcinoma	Resection
4	86	CT abdomen and Pelvis	254	Nonsmall cell carcinoma	Died shortly after definitive diagnosis
5	69	CT colon	136	Bronchoalveolar cell carcinoma	Resection
